# Knowledge, Attitude, and Practice of Metformin Extended-Release Tablets Among Clinicians in China: A Cross-Sectional Survey

**DOI:** 10.3389/fphar.2021.634561

**Published:** 2021-07-12

**Authors:** Chang Liu, Siqi Tang, Kang An, Shengzhao Zhang, Yiling Zhou, Na Su, Rong Yang, Xiaoyang Liao, Zhenmei An, Sheyu Li

**Affiliations:** ^1^ Department of Endocrinology and Metabolism, West China Hospital, Sichuan University, Chengdu, China; ^2^ Department of Cardiology, Peking Union Medical College Hospital, Chinese Academy of Medical Sciences and Peking Union Medical College, Beijing, China; ^3^ Department of Cardiology, West China Hospital, Sichuan University, Chengdu, China; ^4^ Department of General Medicine, West China Hospital, Sichuan University, Chengdu, China; ^5^ Department of Pharmacy, West China Hospital, Sichuan University, Chengdu, China; ^6^ Chinese Evidence-based Medicine Centre, Cochrane China Centre and MAGIC China Centre, West China Hospital, Sichuan University, Chengdu, China; ^7^ Engineering Research Centre of Medical Information Technology, Ministry of Education, West China Hospital, Sichuan University, Chengdu, China

**Keywords:** metformin XR, knowledge—attitude—practice, clinician, metformin immediate release, adherence

## Abstract

**Background:** Metformin extended-release (XR) is a once-daily alternative conventional immediate-release (IR) tablet for adults with type 2 diabetes.

**Aim:** This study aimed to investigate the knowledge, attitude, and practice of the use of metformin XR tablets among clinicians.

**Methods:** We conducted a cross-sectional online survey among endocrinologists, general practitioners, and internists, who are taking routine care of adults with type 2 diabetes in health institutes at all levels in Sichuan Province, China. We designed an online questionnaire including the demographic information, knowledge, attitude, and practice about metformin XR tablets.

**Results:** We included 158 clinicians, 67.7% of whom were females and 63.9% were from tertiary hospitals. The median age was 39.6 years (ranging between 22 and 62 years). Only 8.2% of the clinicians correctly answered the knowledge questions, 82.3% and 62.0% of the responders assumed that metformin XR had superior efficacy and tolerability to the metformin IR, respectively. Only 46.8% of the clinicians prescribed the metformin XR based on the patient’s preference for once daily frequency.

**Conclusion:** The knowledge, attitude, and practice of metformin XR among Chinese clinicians need improving. Clinicians need credible information to support their clinical decision-making regarding metformin XR.

## Introduction

Type 2 diabetes is the leading burdensome chronic disease in China (Li et al., 2018; Li et al., 2020a). Metformin is one of the most frequently prescribed anti-diabetic agents (Inzucchi et al., 2015) and was further demonstrated to be effective in preventing diabetes among the high-risk population by Diabetes Prevention Program (DPP)/DPP Outcomes Study (DPPOS) (Diabetes Prevention Progr, 2015). Over the past two decades, metformin has expanded its role with an array of preparations, notably extended-release (XR), a formulation developed as an alternative to conventional immediate-release (IR) tablets, is intended to be used as part of a once-daily dosing regimen (Fujita and Inagaki, 2017). The metformin XR delivers comparable glycemic control efficacy to IR (Schwartz et al., 2006; Gao et al., 2008; Donnelly et al., 2009; Aggarwal et al., 2018; Ji et al., 2018; Abrilla et al., 2021; Tan et al., 2021) and improves compliance due to the reduced dosing (Schwartz et al., 2006; Aggarwal et al., 2018).

Several studies have suggested that metformin XR is associated with fewer gastrointestinal (GI) adverse events (Gao et al., 2008; Donnelly et al., 2009). However, an international randomized trial in 2017 indicated a similar safety profile between the two formulations (Aggarwal et al., 2018), which was later confirmed by a multi-center study among the Chinese population (Ji et al., 2018). The National Institute for Health and Care Excellence (NICE) guideline recommends metformin IR as the initial drug for type 2 diabetes, and recommends metformin XR for those with GI effects (Type 2 diabetes in adults, 2020).

The XR formulation is characterized by delayed peak plasma concentration occurring 7 h after dosing compared to 3 h for IR, due to the prolonged absorption in the upper gastrointestinal tract after fluid ingestion (Fujita and Inagaki, 2017). These pharmacological properties direct distinct patterns of drug administration. Therefore, the appropriate prescription and instructions from fully informed clinicians are essential for the outcome of glycemic control (Wittich et al., 2014). However, the acceptance rate of professional recommendations and correct dosing strategy from clinicians is unclear. Therefore, we conducted a cross-sectional survey to learn the knowledge, attitude, and practice (KAP) of Chinese clinicians about metformin XR.

## Methods

### Study Design

In this cross-sectional online study, we used a simple random sampling approach to recruit clinicians from the board member of the Sichuan Association of Endocrine and Metabolic Diseases Control and their colleagues (from secondary or tertiary hospitals) and the network of the general medicine in Sichuan (general practitioners from primary care) through the dissemination of the website link. Eligible participants included clinicians who: 1) currently practiced in the registered health care institutes at all levels in Sichuan, China; 2) prescribed metformin and other anti-diabetic drugs independently; 3) consented to the survey; 4) fully engaged in at least ten adults of type 2 diabetes each month and were capable of formulating and adjusting glucose-lowering regimen. We excluded practitioners who held part-time positions in a pharmaceutical company related to the sales of glucose-lowering agents. The online survey was conducted from January 1, 2020, to July 31, 2020. This study was approved by the ethical committee of West China Hospital, Sichuan University (No. 2019-1064).

### Questionnaire Design

The questionnaire was developed and justified by a multi-disciplinary team, including clinical diabetologists, cardiologists, general practitioners, clinical pharmacists, and methodologists. We performed a pilot survey to confirm the feasibility and readability of the questionnaire before the formal survey. The questionnaire consisted of four parts: demographic information (8 questions), including the eligible information of the participants, knowledge (3 questions), attitude (3 questions), practice (4 questions). Questions in the demographic section included sex, age, level of health care institute (primary, secondary, tertiary, others), specialty (endocrinologist, general practitioners, internists, or others), whether affiliated to a teaching hospital, years of practice, cases of treated adults with type 2 diabetes each month, whether they independently prescribe metformin and other anti-diabetic drugs, ways, and personnel of patient instruction. The knowledge section included questions on the dosage range, frequency, and timing of metformin XR. Participants received a 0 point score for an incorrect answer and 1 point score for a correct one. The attitude part included questions of the perceived difference of efficacy and side effects between metformin XR and metformin IR and the favored formulation. The practice part included questions of circumstances, the most important factor for prescribing metformin XR.

### Sample size calculation

We calculated the sample size according to the following formula (Sample Size Calculator (2, 2008),

$$n=\frac{\frac{p\;\times \left(1-p\right)\;\times \;z^{2}}{\varepsilon^{2}}}{1+\frac{p\;\times \left(1-p\right)\;\times \;z^{2}}{N\;\times \;\varepsilon^{2}}}$$

where z is the z score; *ε* is the margin of error; N is the population size; *p* is the population portion.

In our study, we estimated that the total number of general practitioners in Sichuan was 10,394 (5.5% of all general practitioners in China) in 2015 (Wu et al., 2018) with a margin of error, 4% and confidence level, 95%. The sample size was thus 157, conservatively estimating the invalid rate of the questionnaires being 20%.

### Data Analysis

Data were analyzed by SPSS 25.0 (IBM Corporation). Qualitative data are described as frequencies and percentages. Quantitative data are described as median (Interquartile range, IQR). Independent *t*-test or Wilcoxon rank-sum test were used for the comparisons of quantitative data between two groups, and analysis of variance (ANOVA) or nonparametric test was used for the comparisons of quantitative data among three or more groups. Fisher’s exact test was used for examining the association of categorical data. The subgroup analysis for knowledge score included level of health care institute (tertiary, non-tertiary hospital), specialty (endocrinologist, non-endocrinologist), years of practice (<10 years, ≥10 years), cases of treated type 2 diabetes each month (<50 cases, ≥50 cases). All the statistical analyses were two-sided, and *p* < 0.05 was considered statistically significant.

## Results

Among the 216 responders, we excluded 55 responders who treated less than 10 cases of type 2 diabetes each month, and three responders who did not independently make their prescription. The analysis included 158 returned questionnaires. Table 1 shows the demographic characteristics of responders. The mean age of responders was 39.6 ± 7.7 years old (ranged between 22 and 62 years), and 67.7% (107/158) were female. There were 63.9% (101/158) of responders currently practicing in tertiary care institutes, 51.3% (81/158) worked as endocrinologists, 73.4% (116/158) practiced for more than 10 years, and 34.2% (54/158) engaged in more than 50 cases of type 2 diabetes each month. Among the clinicians, 77.8% (123/158) informed patients about medication use in detail, and more clinicians practiced in the non-tertiary hospitals would give detailed instructions (89.5 vs. 71.3%, *p* = 0.01). Furthermore, 72.2% (114/158) and 42.4% (67/158) of the responders expected pharmacists or nurses to implement adult instruction.

**TABLE 1:** Demographic characteristics.

Parameters	N = 158
Age (years)	39.6 ± 7.7
Male sex (%)	51 (32.3)
Level of health care institute	—
Primary	26 (16.5)
Secondary	14 (8.9)
Tertiary	101 (63.9)
Others	17 (10.8)
Affiliated to teaching hospital	94 (59.5)
Year of practice	—
<10 years	42 (26.6)
≥10 years	116 (73.4)
Specialty	—
Endocrinologist	81 (51.3)
Non-endocrinologist*	77 (48.7)
Cases of treated type 2 diabetes each month	—
<50	104 (65.8)
≥50	54 (34.2)
Way of patient instruction	—
In detail	123 (77.8)
In brief	35 (22.2)
Personnel of patient instruction	—
Pharmacists	114 (72.2)
Interns	44 (27.8)
Nurses	67 (42.4)
Others	31 (19.6)

Data are mean ± SD or *n* (%). *Non-endocrinologists include general practitioners, internists, and others.

**FIGURE 1:**
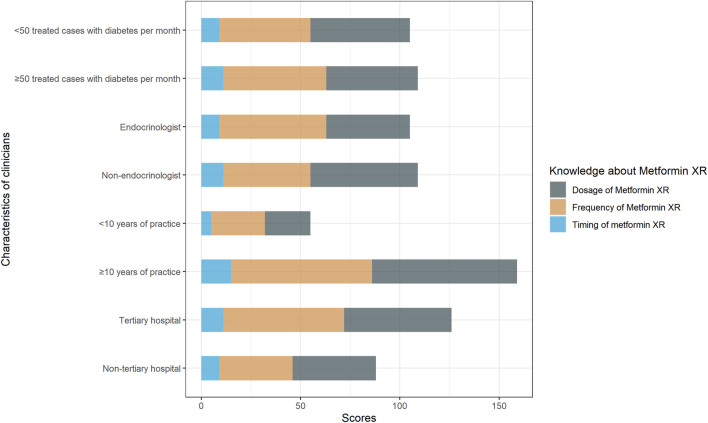
Responses to knowledge questions “the correct dosage range of metformin XR,” “the frequency of metformin XR,” “the proper timing of metformin XR administration.” Responses were compared between clinicians with <10 years of practice and with ≥10 years of practice, endocrinologists and non-endocrinologists, clinicians treated <50 cases and ≥50 cases with type 2 diabetes per month, clinicians practiced in non-tertiary hospital and tertiary hospital.

The median knowledge score was 1 (1, 2). The participants with score 0, 1, 2, and 3 were 16.5% (26/158), 40.5% (64/158), 34.8% (55/158), and 8.2% (13/158), respectively. As shown in Table 2, there were 60.8% (96/158) of the responders chose the correct dose range of metformin XR (500–2000 mg), 62.0% (98/158) chose the correct frequency (once-daily), and 12.7% (20/158) selected the proper timing for drug administration (taking with evening meals). Clinicians practicing in non-tertiary hospitals scored more than those working in tertiary hospitals [2 (1, 2) vs. 1 (1, 2), *p* = 0.03]. Clinicians with more years of practice, working as endocrinologists, and those engaging in more cases of type 2 diabetes did not exhibit better knowledge performance (Figure 1).

**TABLE 2:** Knowledge, attitude and practice.

Knowledge	Median (IQR) or *n* (%) of correct responses
Total score (3)	1 (1, 2)
K1. What is the correct dosage range of metformin XR?	96 (60.8)
K2. What is the correct frequency of metformin XR?	98 (62.0)
K3. What is the proper timing of metformin XR administration?	20 (12.7)
**Attitude**	N (%) of responses
A1. Comparing the metformin IR and XR, which is more effective?	—
The metformin IR	5 (3.2)
The metformin XR	130 (82.3)
Comparable	14 (8.9)
Unclear	9 (5.7)
A2. Comparing the metformin IR and XR, which leads to more adverse effects?	—
The metformin IR	98 (62.0)
The metformin XR	2 (1.3)
Comparable	34 (21.5)
Unclear	24 (15.2)
A3. Which do you prefer to prescribe regarding efficacy and safety?	—
The metformin IR	7 (4.4)
The metformin XR	139 (88.0)
Comparable	7 (4.4)
Unclear	5 (3.2)
**Practice**	N (%) of responses
P1. What are the reasons for choosing the metformin XR?	—
For patients meeting the indications for metformin	136 (86.1)
For patients intolerable to the metformin IR	64 (40.5)
For patients continuously treated with the metformin XR	76 (48.1)
For patients preferring not to take multiple dosing daily	74 (46.8)
Others	5 (3.2)
P2. What is the most important factor for prescribing metformin XR?	—
Safety	57 (36.1)
Efficacy	82 (51.9)
Patients’ preference	5 (3.2)
Price	3 (1.9)
The inventory of pharmacy	11 (7.0)

Data are mean ± SD or *n* (%). XR, extended-release tablets; IR, immediate-release.

**FIGURE 2:**
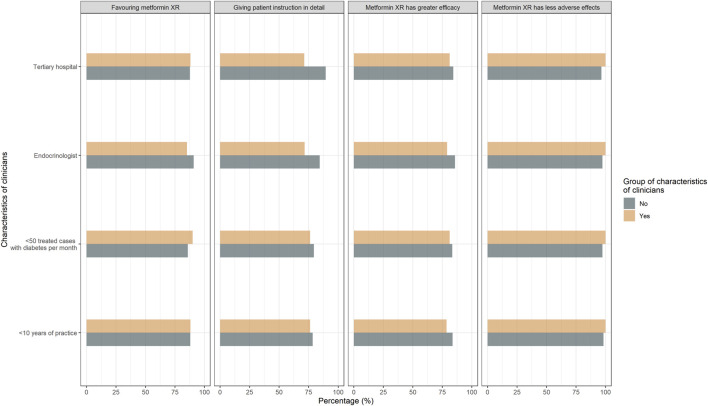
Responses to attitude questions “Comparing the metformin IR and XR, which is more effective?,” “Comparing the metformin IR and XR, which leads to more adverse effects?” “Which do you prefer to prescribe regarding efficacy and safety?” Responses were compared between clinicians with <10 years of practice and with ≥10 years of practice, endocrinologists and non-endocrinologists, clinicians treated <50 cases and ≥50 cases with type 2 diabetes per month, clinicians practiced in non-tertiary hospital and tertiary hospital.

In terms of attitude, 82.3% (130/158) of the responders believed metformin XR was associated with improved efficacy and 62.0% (98/158) believed metformin IR led to greater adverse effects. Only 8.9% (14/158) of the responders acknowledged the similar efficacy profile between the two formulations. Moreover, 88% (139/158) of the participants favored the metformin XR based on efficacy and safety (Table 2). The clinicians’ attitude was not significantly affected by years of practice, the level of the hospital being employed, specialty, and cases of treated type 2 diabetes each week (Figure 2).

**FIGURE 3:**
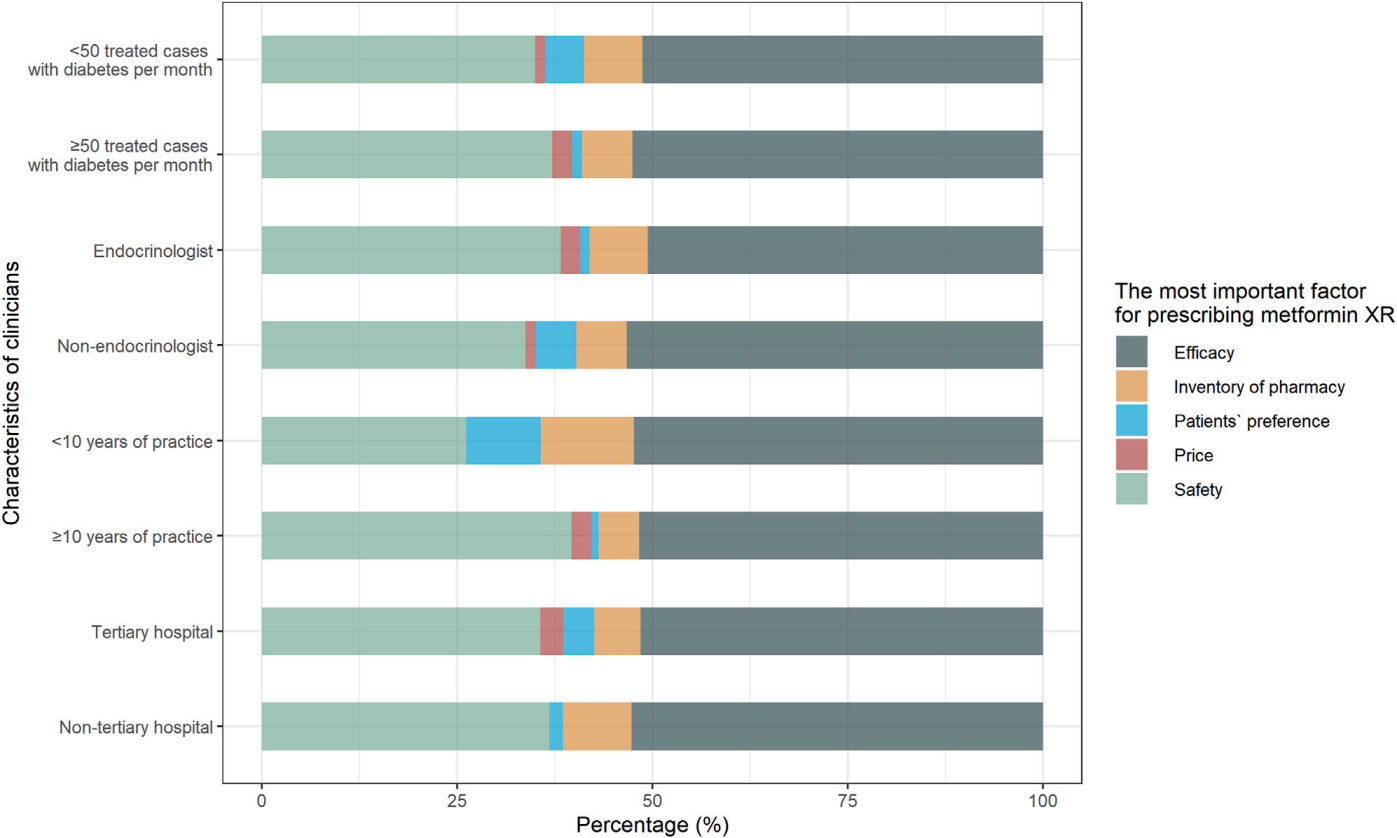
Responses to practice question “What is the most important factor for prescribing metformin XR?” Responses were compared between clinicians with <10 years of practice and with ≥10 years of practice, endocrinologists and non-endocrinologists, clinicians treated <50 cases and ≥50 cases with type 2 diabetes per month, clinicians practiced in non-tertiary hospital and tertiary hospital.

In terms of practice, only 46.8% (74/158) of the responders would suggest the metformin XR for adults who preferred not to take multiple doses daily. Other frequent reasons for prescribing metformin XR were treating those who met the indications for metformin (86.1%, 136/158), and those who were continuously treated with metformin XR (48.1%, 76/158). When prescribing metformin in different formulations, only 3.2% (5/158) of the responders prioritized patient preference while the majority attributed it to the assumed better efficacy (51.9%, 82/158) or safety (36.1%, 57/158). Clinicians with less than 10 years of practice considered the patients’ preference (9.5 vs. 0.9%) and the inventory of pharmacy (11.9 vs. 5.2%) more (*p* = 0.02) (Figure 3).

## Discussion

To the best of our knowledge, this is the first KAP study of metformin XR in clinicians. We demonstrated the marked insufficiency of the KAP toward this relatively novel formulation across different types of practitioners from different levels of health care institutes in China.

Good adherence to metformin improves clinical outcomes (Nathan et al., 2006; Patel et al., 2019; Wu et al., 2018; Walker et al., 2020). Randomized controlled trials and systematic reviews have demonstrated that the XR formulation improves drug adherence by 10% (Gao et al., 2008) because of its simpler usage (Schwartz et al., 2006; Aggarwal et al., 2018; Ji et al., 2018; Abrilla et al., 2021; Tan et al., 2021). However, in our survey, nearly 40% of the clinicians overlooked this advantage. Over 80% prescribed metformin XR because of this effectiveness, which was not backed by the latest evidence (Schwartz et al., 2006; Gao et al., 2008; Donnelly et al., 2009; Aggarwal et al., 2018; Ji et al., 2018; Abrilla et al., 2021; Tan et al., 2021). Our results suggest that a misconception of the features of metformin XR is common among Chinese clinicians, which may lead to inappropriate prescription and fluctuation of long-term glucose control (Li et al., 2020b). There is a clear gap in evidence dissemination from literature to practice in China. It is noteworthy that clinicians from tertiary hospitals, who take in major charge of evidence dissemination in the country, performed no better than those from primary care or secondary hospitals. This calls for evidence-based continuous education of clinicians and prescription audits by clinical pharmacists at all levels of Chinese healthcare institutes (Donnelly et al., 2009; Xin et al., 2014). Unfortunately, our study showed that over 1/4 of the clinicians did not receive help or guidance from clinical pharmacists.

Less than 2/3 of the clinicians correctly respond to the timing of the drug, according to the drug label (® Extended-Releas, 2017; ® Tablets and G, 2017), which is essential for the drug to reach its optimal glycemic-control effect (Sato et al., 2019). The findings also indicate the potential inappropriate practice of metformin IR use, which call for future investigation. Our current study suggests a similar insufficiency of KAP of metformin XR among clinicians compared to adults living with type 2 diabetes, based on our previous findings (Liu et al., 2021). The present study indicates that evidence dissemination strategy may warrant novel techniques and structured re-organization to systematically improve the practical performance of both healthcare providers and patients (Wu et al., 2017; Contreras and Vehi, 2018; Wu et al., 2019). Structuralized guidelines and the clinical decision support system (CDSS) may improve this condition via electronic health record systems without adding work or study load for clinicians (Siemieniuk et al., 2016). This study has also noted a potential lack of direction when adapting international evidence to local settings (Zhou et al., 2021).

### Limitation

Our study has some limitations. Firstly, it focuses only on clinicians from Sichuan, China. The situation can be different in other countries and other provinces in China, and need external validation in other regions and countries. However, our study unexpectedly showed a clear gap in the knowledge of clinicians managing adults with type 2 diabetes using metformin XR. Secondly, we cannot access the response rate because of the sampling approach. Despite these limitations, our study covers clinicians from health care institutes at all levels and did not show the result-changing subgroup effect of the answers.

## Conclusion

Our study identified a clear gap in the knowledge, attitude, and practice of the metformin XR among Chinese clinicians. The clinicians need to improve their practice by receiving credible information to support clinical decision-making regarding metformin XR. Novel techniques and evidence dissemination strategies may help improve the condition without adding to the work or study load of clinicians.

## Data Availability

The original contribution presented in the study are included in the article/Supplementary Material, further inquiries can be directed to the corresponding author.
